# Management of infants with congenital diaphragmatic hernia and pulmonary hypertension—one size does not fit all

**DOI:** 10.1038/s41390-023-02551-z

**Published:** 2023-03-02

**Authors:** Anne Greenough

**Affiliations:** Department of Women and Children’s Health, School of Life Course Sciences, Faculty of Life Sciences and Medicine, King’s College London, London, UK

Congenital diaphragmatic hernia (CDH) occurs in approximately 1 in 3000 live births. It is associated with a mortality rate of approximately 30% due to pulmonary hypoplasia and pulmonary hypertension (PH). Analysis of data from the CDH study group registry demonstrated that 86.5% of 1472 infants who had an echocardiogram in the first 48 h after birth had PH.^1^ PH was independently associated with an increased risk of mortality or oxygen support at 30 days and utilisation of extracorporeal life support (ECLS). Inhaled nitric oxide (iNO), a pulmonary vasodilator, is then frequently given to CDH infants. Yet, the evidence for such a strategy is weak with a number of studies showing no overall positive effects. The NINOS-CDH multicentre randomised controlled trial (RCT), which included 53 infants, failed to demonstrate a reduction in the combined outcome of ECLS use and mortality in infants who received 20 ppm of iNO compared to those who received 100% oxygen. Indeed, the use of ECLS was significantly higher in the iNO group suggesting iNO given early in CDH infants was disadvantageous.^2^ Furthermore, two large observational studies demonstrated no benefit, but significant healthcare costs.^3,4^ In other studies, some infants have had benefits. In one, 38 of 95 infants treated with iNO had a positive response in oxygenation and were less likely to require ECLS,^5^ and in another, 28 of 72 infants with a positive response were more likely to survive.^6^ The likely reason for this differential effect is that the PH seen in CDH infants is complex and iNO only addresses certain components. Neonates with CDH and PH have pulmonary vasoconstriction and ventilation-perfusion mismatching, which should respond to iNO. They, however, are also at risk of left ventricular hypoplasia due to in utero compression of the left ventricle (LV) by abdominal contents as well as alteration in LV filling haemodynamics. In such patients, iNO has the potential to worsen pulmonary venous hypertension.

A possible way then to identify those likely to respond would be to perform echocardiographic examinations before considering starting iNO. In the study reported in *Pediatric Research*,^7^ data collected between 2015 and 2020 by the CDH Study Group was explored to test the hypothesis that infants with echocardiographic characteristics of severe PH with right ventricular dysfunction might benefit from iNO, but iNO might worsen those with severe PH and impaired left ventricular function. Echocardiographic findings were related to the impact of early iNO use in the first 3 days after birth on mortality or ECLS. Of 1777 infants, 863 received early iNO treatment; they had a lower birth weight, larger defect size, more severe PH and abnormal ventricular size and function. After controlling for those factors, early iNO use was associated with increased mortality and ECLS use. After stratification by echocardiographic characteristics and defect size, no subgroup had a reduction in mortality or ECLS use.^7^ The authors postulated that the lack of efficacy even in the absence of LV dysfunction might have been due to aberrant pulmonary vascular development, blunted NO signalling pathway and/or over-expression of endothelin (ET)-1 and ETA, a mediator of vasoconstriction. The strengths of the study are that a large number of infants were included and subgroup analysis was undertaken by echocardiographic criteria. There was, however, no standardisation regarding echocardiographic techniques and only data from a subset of the total population were analysed. The dosage of iNO was not given. Importantly, the response to inotropes was not collected and, given the causations of PH in CDH infants, it is likely a combination of treatments that may be needed to treat it successfully.

Due to abnormal pulmonary development, CDH infants who are most likely to die can be identified in the delivery suite, as they were less likely to respond to resuscitation due to stiffer lungs and reduced pulmonary vasodilation.^8^ Thus, CDH infants may benefit from antenatal interventions. The most successful has been fetoscopic endoluminal tracheal occlusion (FETO). In foetuses with isolated left-sided severe CDH, FETO performed at 27–29 weeks of gestation resulted in significant benefit over expectant care with respect to survival to discharge.^9^ As FETO is associated with increased risks of preterm, prelabour rupture of membranes and preterm labour, it was performed in infants with moderate left CDH with FETO only at 30–32 weeks. In that group, there was no significant increase in survival to discharge or the need for supplemental oxygen at 6 months, which may reflect that the duration of balloon occlusion was too short.^10^ Foetal lung growth is stimulated by tracheal occlusion; it is important to determine in FETO-treated infants whether iNO is more effective and if this is related to less LV dysfunction.

There are many strategies to treat PH in CDH infants and these should be assessed in appropriately designed RCTs with sufficient numbers to determine if there was effectiveness with regard to clinically important outcomes for the infants, their parents and healthcare practitioners. The CDH Euro Consortium was set up to facilitate such trials, but recruitment was terminated in an RCT of different modes of respiratory support due to slow recruitment. Nevertheless, it was still possible to demonstrate that infants supported by conventional ventilation required fewer ventilator days, less often needed extracorporeal membrane oxygenation and received iNO, sildenafil or vasoactive drugs.^11^ As a consequence, the CDH EURO consortium update of 2015 recommended conventional mechanical ventilation as the optimal initial ventilation strategy,^12^ but that does beg the question of which conventional mode? More recently, the CoDiNOS international trial is investigating whether intravenous sildenafil or iNO is better at improving outcomes in PH in infants with CDH.^13^ Recruitment to that trial, however, was also challenging, not least due to differences in regulatory requirements across European countries. Nevertheless, we should continue to try to undertake RCTs and, as a community, seek ways to improve recruitment. Registries are also an important resource, but it is important that the data collection is comprehensive and methods which make this less burdensome should be identified.

In conclusion, iNO should not be started in CDH infants without echocardiographic evidence that it might be efficacious and then the response should be carefully monitored with a readiness to stop if the expected improvements do not occur. A personalised approach needs to be taken to the management of PH in CDH infants.
